# Integrating tobacco control into China's health management: Strategies for healthier China

**DOI:** 10.1016/j.pccm.2024.08.005

**Published:** 2024-10-18

**Authors:** Dan Xiao, Chen Wang

**Affiliations:** a Department of Tobacco Control and Prevention of Respiratory Disease, China Japan Friendship Hospital, WHO Collaborating Center for Tobacco Cessation and Respiratory Diseases Prevention, National Respiratory Medical Center, China National Clinical Research Center for Respiratory Diseases, Institute of Respiratory Medicine, Chinese Academy of Medical Sciences, Beijing 100029, China; b Chinese Academy of Medical Sciences and Peking Union Medical College, Beijing 100730, China

Tobacco smoking is a leading global preventable health risk. Smoking can damage nearly all the organs in the body and is recognized as a “common risk factor” for major chronic diseases, such as chronic respiratory diseases, cancer, cardiovascular diseases, and diabetes.^1^^,^^2^

China is the world's largest producer and consumer of tobacco, and also the country most affected by its impact. In 2020, the prevalence of current smokers among individuals aged ≥15 years in China was 25.8%.^3^ A large-scale prospective cohort study of China Kadoorie Biobank involving 512,000 Chinese adults showed that smoking was associated with higher risks of 22 causes of death (17 for men and 9 for women) and 56 individual diseases (50 for men and 24 for women) across all body systems. Compared with people who had never smoked, the risk of developing any disease was approximately 10% higher for men who had ever smoked regularly (hazard ratio [HR]: 1.09, 95% confidence interval [CI]: 1.08–1.11), ranging from 6% for diabetes (HR: 1.06, 95% CI: 1.02–1.09) to 216% for larynx cancer (HR: 3.16, 95% CI: 1.98–5.05). Men also experienced significantly more frequent and longer stays in hospital, particularly due to cancers and respiratory diseases.^4^ Smoking also significantly increases the risk of various non-directly fatal diseases such as asthma, peptic ulcers, cataracts, diabetes, and other metabolic diseases. These health hazards mean that tobacco smoking is estimated to account for more than one million deaths each year in China, surpassing the total number of deaths from human immunodeficiency virus (HIV)/acquired immunodeficiency syndrome (AIDS), tuberculosis, traffic accidents, and suicide combined. Without effective action to drastically reduce the smoking prevalence, this number is projected to increase to two million deaths annually by 2030 and three million deaths annually by 2050.^5^ Comprehensive tobacco control efforts, including policy changes and public health initiatives, are essential for achieving the Healthy China 2030 goals, emphasizing that sustained reductions in smoking prevalence are critical for improving overall public health and reducing tobacco-related health disparities.^6^

Over time, the focus on supporting health and longevity has been increasing. This article therefore aims to explore how to integrate tobacco control into China's health management system to better improve health and quality of life among the population.

## The need to integrate tobacco control into China's health management system

The pervasive nature of tobacco smoking and its profound detrimental effects mean that it is imperative to integrate comprehensive tobacco control measures into health management strategies. First, incorporating tobacco control into health management strategies should help to mitigate the poor health consequences of smoking. Reducing smoking prevalence should safeguard the collective well-being of the populace and also reduce the strain on health care systems.^7^ Preventive measures and cessation programs can also reduce the burden of health care expenditure, increasing the sustainability of health care infrastructure.

Second, the integration of tobacco control into China's health management framework is essential for social and economic development. Beyond its direct impact on physical health, smoking exerts a profound toll on societal productivity and economic prosperity.^8^ Instances of absenteeism due to smoking-related illnesses and the need for extensive medical treatment reduce individual productivity and translate into substantial economic losses on a macroeconomic scale. Health management initiatives that comprehensively address tobacco consumption can help to encourage better health and greater productivity, driving societal progress.

In addition to the tangible health and economic benefits, the integration of tobacco control measures into health management strategies can be helpful in increasing public awareness and self-protection consciousness.^9^ Targeted educational campaigns and awareness-raising initiatives can provide individuals with the knowledge and tools necessary to make informed decisions about tobacco use. These approaches can show the stark realities of tobacco-related harm and foster a culture of self-care and responsibility. They may therefore catalyze transformative shifts in societal attitudes toward tobacco consumption.

Finally, by promoting tobacco cessation and advocating healthy lifestyles, integrated tobacco control measures can contribute to the broader goal of enhancing overall public health outcomes. These initiatives can enable individuals to move on from tobacco addiction and embrace healthier habits, improving health and well-being across society.

The integration of tobacco control into health management strategies is therefore a multifaceted approach with far-reaching implications. It addresses the complex interplay of health, economic, and societal factors associated with tobacco consumption, and holds potential to usher in a new era of health and prosperity for communities.

## Feasible measures to integrate tobacco control into health management

### Embracing smoking cessation as a cornerstone of comprehensive tobacco control strategies

In the intricate landscape of tobacco control, a pivotal aspect is promoting smoking cessation. This is complex and enduring, and requires a deep understanding of the interplay between individuals’ physiological predispositions, psychological inclinations, and environmental influences. It is therefore essential to establish a robust framework for smoking cessation and tobacco dependence treatment.

First, specialized knowledge and skills in tobacco control, particularly smoking cessation, are essential. Smoking cessation interventions should be grounded in scientific evidence and clinical practice, emphasizing holistic care, personalized approaches, and ongoing support. Currently, several evidence-based smoking cessation strategies exist. These include psychological interventions, behavioral therapies, and medication. Smoking cessation clinics provide intensified support for individuals trying to stop smoking. Health care providers must integrate smoking cessation services into routine clinical practice to ensure equitable access to effective cessation support. Digital interventions such as “mCessation,” which is delivered through the WeChat platform, can offer tailored cessation messages over time. A real-world study showed that this project had a smoking cessation success rate of 21.9%, meaning approximately one in five participating smokers successfully quit smoking supported by “mCessation.”^10^ This rate of 21.9% was higher than that in the Indian study (19%) and the Veteran's study (6.7%).^11^^,^^12^ Efforts should be made to promote this project to reach more smokers and expand its coverage.

The training and oversight of tobacco control professionals should also be strengthened. Comprehensive training programs should cover foundational principles of tobacco control and smoking cessation strategies. Standardized protocols should be implemented, including thorough smoking history assessments and seamless integration of tobacco dependence treatment into clinical workflows. Vigorous monitoring and evaluation mechanisms enable stakeholders to identify areas for improvement. Tobacco control professionals need medical and psychological expertise, as well as accountability and communication skills. They play a vital role in delivering comprehensive cessation interventions, supporting individuals to stop smoking, and empowering them to overcome tobacco dependence.

Finally, it is important to advocate for policies and regulations on smoking cessation. Efforts should focus on bolstering the accessibility, affordability, and availability of cessation services, supported by robust policy frameworks and legal safeguards.

### Raising public awareness of tobacco dependence as the primary cause of long-term smoking, inherently a fatal chronic disease

Most smokers are aware of the health hazards of smoking, but find that quitting is not easy. This is primarily because of nicotine, the highly addictive substance in tobacco.^13^ Nicotine is a psychoactive substance, and when inhaled by smokers, it rapidly enters the bloodstream through the lungs and reaches the central nervous system within seconds. It acts on nicotine receptors in the brain, producing a sense of pleasure, temporarily improving work performance, cognitive abilities, extending attention span, and alleviating anxiety and depression.

The long-term effects of nicotine include significant changes in the brain structure of smokers. In particular, gray and white matter atrophy worsens with increased smoking. Brain function also undergoes significant changes with abnormal resting-state neural activity in the frontal, temporal, and parietal lobes and significantly reduced functional connectivity between insula and orbitofrontal cortex, superior frontal gyrus, and temporal lobe.^14^, ^15^, ^16^

Smoking causes addiction, and we therefore talk about tobacco dependence. As early as 1980, the American Psychiatric Association listed tobacco dependence as an independent disease in the Diagnostic and Statistical Manual of Mental Disorders (DSM-3).^17^ The World Health Organization's International Classification of Diseases (ICD) considers it a substance use disorder, coded as F 17.2.^18^

The *Chinese Clinical Guideline for Smoking Cessation (2015 Edition)*^19^ clearly defines the diagnostic criteria for tobacco dependence. Diagnosis can be made if at least three of the following six criteria have been met within the past year: (1) a strong craving or desire to use tobacco; (2) unsuccessful efforts to cut down or control tobacco use; (3) experiencing tobacco withdrawal symptoms (such as irritability, frustration, anger, anxiety, difficulty in concentrating, increased appetite, restlessness, and insomnia) after abrupt cessation of tobacco use, or reduction in the amount of tobacco used; (4) tolerance of tobacco, defined as the need for markedly increased amounts of tobacco to achieve the desired effect; (5) having given up or reduced important social, occupational, or recreational activities because of tobacco use; and (6) continuing tobacco use despite a persistent or recurrent physical or psychological problem that is likely to have been caused or exacerbated by tobacco. The severity of tobacco dependence can be assessed using the Fagerström Test for Nicotine Dependence (FTND)^20^ and the Heaviness of Smoking Index (HSI).^21^ A higher cumulative score on these scales shows more severe tobacco dependence.

The 2018 China Health Literacy Survey (CHLS), the world's first epidemiological survey on tobacco dependence based on a nationally representative population, found that the prevalence of tobacco dependence among current smokers was as high as 49.7%, with nearly half of smokers being dependent on tobacco. There were no significant differences between genders or urban and rural areas. On this basis, it is estimated that approximately 183.5 million current smokers in China in 2018 were dependent on tobacco, of whom 177.5 million were male.^22^ Non-tobacco-dependent smokers have a 2.88 times higher likelihood of successfully quitting smoking than tobacco-dependent smokers. There are regional differences in the prevalence of tobacco dependence, with Beijing, Shanghai, and Zhejiang having the lowest rates, and Qinghai, Shandong, Jiangxi, and Xinjiang having the highest rates.

### Strengthening publicity and education to raise population health awareness

Strengthening publicity and education is also crucial, as it enables individuals to understand the reality and severity of tobacco hazards, increases public health awareness, and promotes overall awareness of tobacco control. Publicity and education can take various forms, including advertisements, public service announcements, WeChat promotion, knowledge lectures, and health education courses. These approaches can highlight a wide range of information, including the reality of tobacco hazards, the need for tobacco control, tobacco control policies and measures, and smoking cessation methods and techniques. The form and content of publicity and education should be adjusted and optimized to fit different target audiences and situations to achieve the best possible effect. In publicity and education, attention should be paid to the following points.

#### Targeting different populations

People of different ages, genders, occupations, and educational levels have different levels of understanding of the hazards of tobacco. Publicity and education should therefore be tailored to target populations. For example, for adolescents and minors, emphasis should be placed on the harmful effects of smoking on physical and intellectual development. It is also helpful to provide positive lifestyle and recreational choices to encourage young people to maintain a healthy lifestyle. Tobacco-dependent individuals need information on and techniques for smoking cessation, and how to seek treatment.

#### Based on scientific evidence

Publicity and education should be consistent with scientific principles, convey truthful information, and avoid exaggerating or downplaying the hazards of tobacco to prevent misleading the public.^23^

#### Emphasizing diversity

Publicity and education should use various forms to avoid reliance on a single form or content. This will help to ensure its acceptance and understanding by the public.

## Integrating tobacco control into health management systems

Tobacco smoking is the primary cause of many diseases, and tobacco control is the primary means of prevention. We should therefore strive to increase public awareness of the hazards of smoking, especially recognizing that “tobacco dependence is a fatal, chronic, highly recurrent, and addictive disease”.^23^

As with the health promotion, prevention, diagnosis, and treatment of other chronic non-communicable diseases, governments, hospitals, professional institutions, and communities should provide support, integrate smoking cessation and tobacco dependence treatment into routine clinical treatment, and improve relevant systems. This will help to develop new therapies for smoking cessation and tobacco dependence treatment, improving the prevention and control of smoking-related chronic diseases.

Long-term management models that are suitable for smoking cessation and tobacco dependence treatment among the Chinese population should also be actively explored. It is essential to reduce the burden of smoking-related non-communicable diseases in China, promote the development of the “big health” industry, and contribute to the development of “Healthy China”.

In conclusion, integrating tobacco control into China's health management system is a long-term and arduous task but essential to build a healthy China. It will need work across society, but we believe that effective policy measures and comprehensive health interventions can reduce the harm caused by smoking to individuals’ physical health and social development, lay a solid foundation for building a healthy China, and make positive contributions to health, families, and society. We should work together to advocate for healthy lifestyles, avoiding tobacco smoking, and contributing to overall population health.

## Funding

This work was funded by the National High Level Hospital Clinical Research Funding (No. 2022-NHLHCRF-LX-01-0102).

## Declaration of competing interest

None.

## References

[1] China Ministry of Health (2012).

[2] China Health Commission. China's Report on the Health Hazards of Smoking 2020. Beijing: People's Medical Publishing House, 2021.

[3] Yan YF, Lin BL, Xu QQ (2023). Utilization of smoking cessation support among adults: 18 PLADs, China, 2020. China CDC Wkly.

[4] Chan KH, Wright N, Xiao D (2022). Tobacco smoking and risks of more than 470 diseases in China: a prospective cohort study. Lancet Public Health.

[5] Chen Z, Peto R, Zhou M (2015). Contrasting male and female trends in tobacco-attributed mortality in China: evidence from successive nationwide prospective cohort studies. Lancet.

[6] Goodchild M, Zheng R (2019). Tobacco control and Healthy China 2030. Tob Control..

[7] Peng W, Chen S, Chen X (2023). Trends in major non-communicable diseases and related risk factors in China 2002-2019: an analysis of nationally representative survey data. Lancet Reg Health West Pac.

[8] Chan KH, Xiao D, Zhou M, Peto R, Chen Z. (2023). Tobacco control in China. Lancet Public Health.

[9] Britton J, Bogdanovica I. (2013). Tobacco control efforts in Europe. Lancet.

[10] Su Z, Wei X, Cheng A (2023). Utilization and effectiveness of a message-based tobacco cessation program (mCessation) in the Chinese general population: longitudinal, real-world study. J Med Internet Res.

[11] Gopinathan P, Kaur J, Joshi S (2018). Self-reported quit rates and quit attempts among subscribers of a mobile text messaging-based tobacco cessation programme in India. BMJ Innov.

[12] Christofferson DE, Dennis PA, Hertzberg JS (2021). Real-world utilization and outcomes of the Veterans Health Administration's Smoking Cessation Text Message Program. Nicotine Tob Res.

[13] Benowitz NL. (2010). Nicotine addiction. N Engl J Med.

[14] Wang S, Zuo L, Jiang T, Peng P, Chu S, Xiao D. (2017). Abnormal white matter microstructure among early adulthood smokers: a tract-based spatial statistics study. Neurol Res.

[15] Zhou S, Xiao D, Peng P (2017). Effect of smoking on resting-state functional connectivity in smokers: an fMRI study. Respirology.

[16] Peng P, Wang Z, Jiang T, Chu S, Wang S, Xiao D (2017). Brain-volume changes in young and middle-aged smokers: a DARTEL-based voxel-based morphometry study. Clin Respir J.

[17] *American Psychiatric Association. Diagnostic and Statistical Manual of Mental Disorders* (1980).

[18] World Health Organization (1992).

[19] China National Health and Family Planning Commission (2015).

[20] Heatherton TF, Kozlowski LT, Frecker RC, Fagerström KO. (1991). The Fagerström test for nicotine dependence: a revision of the Fagerström Tolerance Questionnaire. Br J Addict.

[21] Lim KH, Cheong YL, Sulaiman N (2022). Agreement between the Fagerström test for nicotine dependence (FTND) and the heaviness of smoking index (HSI) for assessing the intensity of nicotine dependence among daily smokers. Tob Induc Dis.

[22] Liu Z, Li YH, Cui ZY (2022). Prevalence of tobacco dependence and associated factors in China: findings from nationwide China Health Literacy Survey during 2018-19. Lancet Reg Health West Pac.

[23] Xiao D, Wang C. (2019). Tobacco dependence should be recognised as a lethal non-communicable disease. BMJ.

